# Unexpected response to palliative radiotherapy for subcutaneous metastases of an advanced small cell pancreatic neuroendocrine carcinoma: a case report of two different radiation schedules

**DOI:** 10.1186/s12885-020-06845-x

**Published:** 2020-04-15

**Authors:** Maria Paola Ciliberti, Roberta Carbonara, Antonietta Grillo, Anna Maria Leo, Ivan Lolli, Carmela Ostuni, Laura Troiani, Barbara Turi, Simona Vallarelli, Angela Sardaro

**Affiliations:** 1 Radiation Oncology Unit, Azienda Ospedaliero-Universitaria Policlinico, P.zza Giulio Cesare nr.11, 70124 Bari, Italy; 2 Interdisciplinary Department of Medicine, Section of Radiology and Radiation Oncology, University of Bari “Aldo Moro”, P.zza Giulio Cesare nr.11, 70124 Bari, Italy; 3 Department of Oncology, National Institute of Gastroenterology “Saverio De Bellis”, Research Hospital, Via Turi, 27 Castellana Grotte, Bari, Italy

**Keywords:** Palliative radiotherapy, Subcutaneous metastases, Pancreatic neuroendocrine carcinoma

## Abstract

**Background:**

Skin metastases from pancreatic neuroendocrine carcinoma (PNEC) are rare and their palliative treatment is challenging. We report our experience in the multimodal management of one of the few reported cases of metastatic PNEC with multiple visceral and subcutaneous secondary lesions, focusing on the effectiveness of palliative radiotherapy for skin metastases.

**Case presentation:**

A 61-years old woman affected by a metastatic PNEC – with subcutaneous growing and bleeding secondary lesions (at the scalp, right scapular region and at the back of the left thoracic wall, respectively) – obtained a successful control of visceral metastases with the use of chemotherapy and an unexpected local response of her skin metastases with palliative radiotherapy. In particular, two subsequent radiation treatments were performed using different fractionation schedules (30 Gy in 10 fractions and 20 Gy in 5 fractions, respectively). Both radiation treatments were well-tolerated and patient’s quality of life was improved. Local response was maintained until patient’s death – that occurred due to cachexia.

**Conclusions:**

The presented case highlights the effectiveness and the good tolerance of radiotherapy in the treatment of subcutaneous metastases; nevertheless, further knowledge of the optimal local palliative approach for PNEC metastatic sites is necessary. The experience gained in this work is the occasion to encourage a routine integrated multidisciplinary team management of metastatic PNECs because of their clinical complexity. The aim is to guarantee the optimization of the care with personalized and more effective systemic and local treatments – also including supportive cares and treatment-related side effects management.

## Background

Pancreatic neuroendocrine tumors (PNETs) are a rare heterogeneous group of neoplasms [1] that have been classified into two broad categories based on the combination of morphological features and proliferation grading system (G), assessed by the Ki-67 index [1]: well-differentiated PNET G1 (Ki-67 index ≤2%) and G2 (Ki-67 index: 3–20%) and poorly differentiated pancreatic neuroendocrine carcinoma PNEC - G3 (Ki-67 > 20%). Currently, the revised gastroenteropancreatic (GEP)-NET classification acknowledges well differentiated Grade 3 NETs in addition to grade 3 poorly differentiated NEC based on morphology [1]. Patients with poorly differentiated PNEC may be further stratified on the basis of whether their Ki-67 index is between 20 and 55% or is > 55%. Patients with a Ki-67 index > 55% have a worse prognosis and may quickly progress to an unresectable disease with symptoms of mass effects, distant metastases (usually hepatic) or both.

Skin metastases from neuroendocrine tumors are very rare [2–4]. Their presence is sign of poor prognosis and their local management can represent a challenge in the case of rapidly growing lesions which tend to ulcerate and to bleed.

We describe a singular case of multiple subcutaneous metastases from a pancreatic neuroendocrine carcinoma which gained an unexpected response to palliative radiotherapy.

## Case presentation

A 61-year-old woman presented with upper abdominal pain steady and radiating to the back and a 2 months history of diarrhea, weight loss and fatigue. Physical examination revealed abdominal distension and moderate epigastric pain. Furthermore, the patient presented multiple subcutaneous nodules on the scalp and chest. The nodules were well-circumscribed, skin- or pink-colored, painless to the touch, with a hardened consistency and no adherence to deeper planes, measuring about 1 cm.

**Fig. 1:**
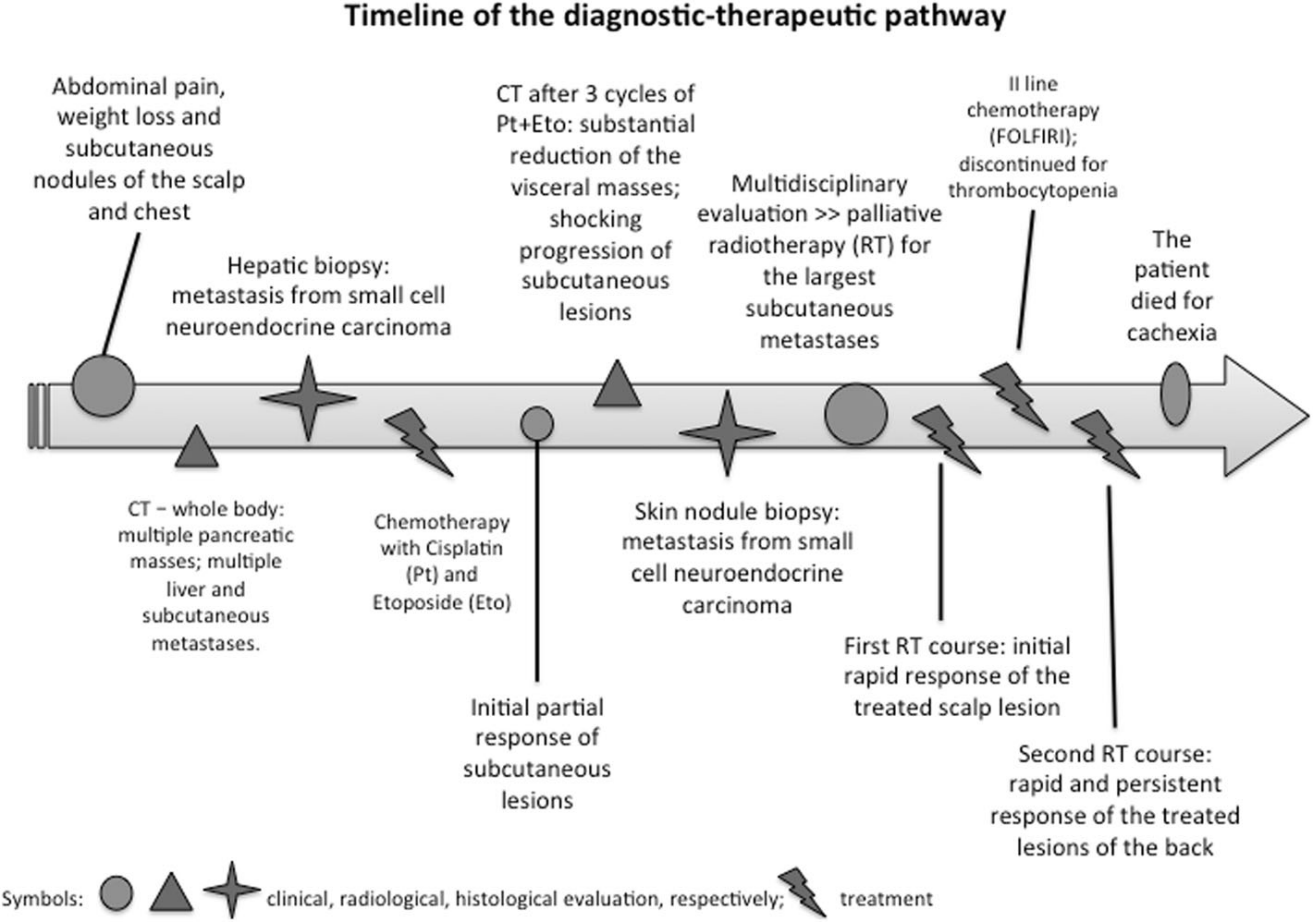
Timeline of the diagnostic-therapeutic pathway

Figure 1 represents a timeline of the diagnostic and therapeutic pathway.

A full-body computer tomography (CT) showed multiple large heterogeneous enhancing masses at the whole pancreas, sized from 4 to 6 cm, and multiple liver metastases – the largest of about 10 cm at the IV-VIII hepatic segment. The CT also confirmed the presence of multiple enhancing subcutaneous metastases.

An ultrasound-guided percutaneous biopsy of the largest hepatic metastasis showed a massive infiltration of the hepatic parenchyma by a solid neoplasm of poorly differentiated monomorphic elements with scarce cytoplasm and focal trabecular aspects. Immunohistochemical investigation was positive for Synaptophysin and CD56, negative for Chromogranin A and Neuron Specific Enolase (NSE) and was consistent with the diagnosis of metastasis of NEC/small cell carcinoma with Ki-67 90% as additional poor prognostic factor.

**Fig. 2:**
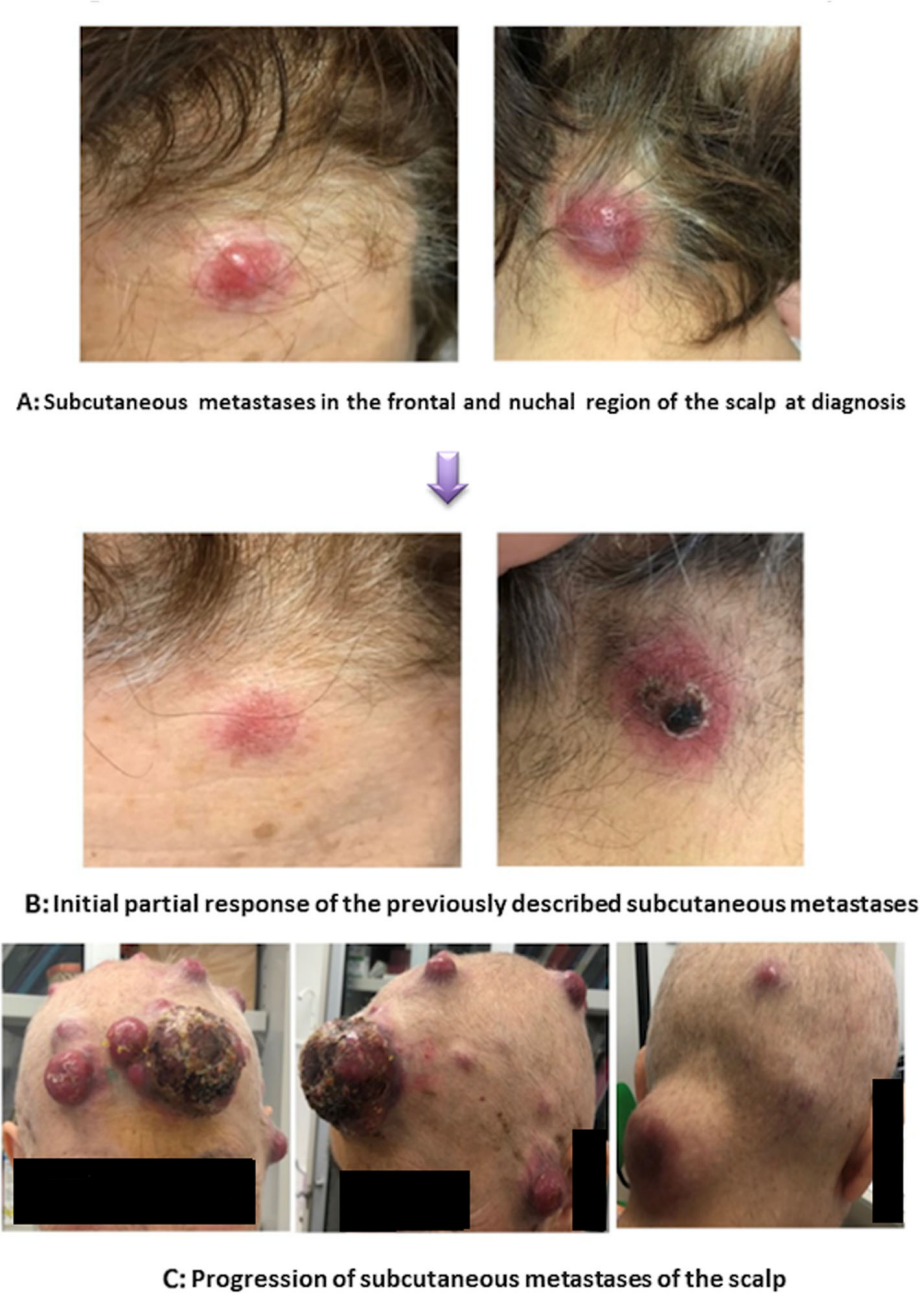
Clinical evaluation of subcutaneous metastases of the scalp

After the Medical Oncologist evaluation, the patient started a platinum based chemotherapy with 6 cycles of Cisplatin and Etoposide. Clinically, after an initial partial response (Fig. 2a-b), the subcutaneous nodules had a shocking rapid progression during chemotherapy (Fig. 2c).

Some of these nodules reached the size of more than 10 cm, with tendency to skin ulceration and bleeding; others were only purple-colored without ulceration. Furthermore, new peri-centimetric nodules appeared at the skin of both legs, but they were palpable more than visible. All the nodules were painless and not adherent to deeper planes.

**Fig. 3:**
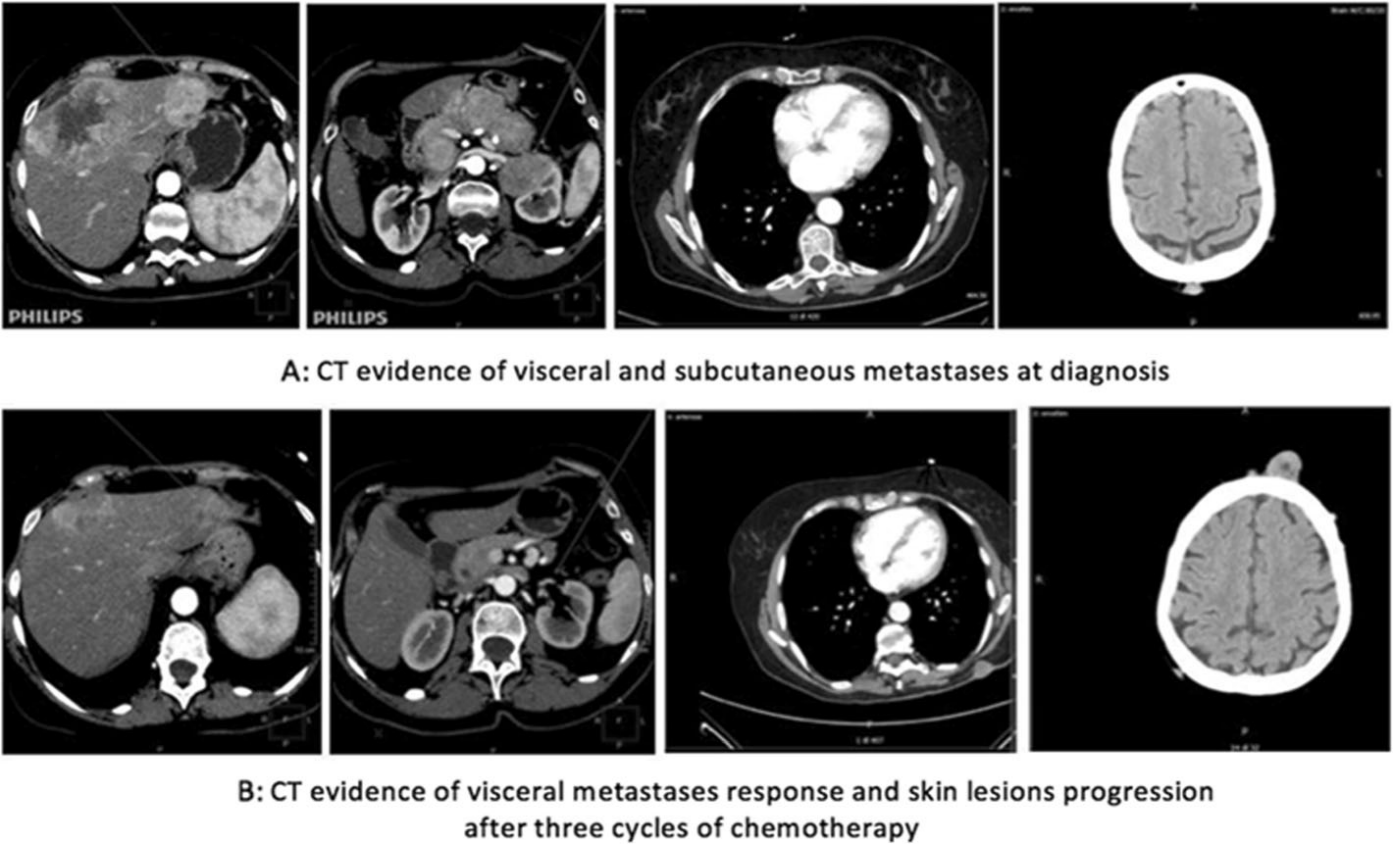
Changes in visceral and skin metastases detected by CT images

Unexpectedly, the control CT performed after three cycles (Fig. 3b) revealed a substantial reduction of the visceral masses while confirming skin metastases progression.

A combined 18F-FDG CT/PET fusion images showed pathological uptake value in the previously mentioned disease sites. After 6 cycles of chemotherapy, taking into account the different response of visceral masses and skin lesions, a biopsy of a skin nodule was performed: it confirmed that subcutaneous nodules were metastases from the primary small cell neuroendocrine carcinoma with positivity for Synaptophysin and CD56 and negativity for Chromogranin A and CK20. Nuclear staining of ki-67 was positive and the ki-67 index reached 90%. After a multidisciplinary evaluation, the patient was referred to a Radiation Oncologist for a palliative treatment of the largest subcutaneous metastases.

**Fig. 4:**
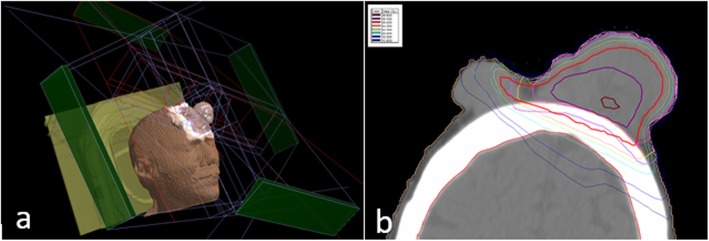
Three-dimensional view of the treatment plan (**a**) and treatment planning dose distribution in axial view (**b**) for a subcutaneous metastasis of the scalp. Four tangential beams were used. Planning was performed through a 3D-conformal technique

At the moment of the first physical examination performed by the Radiation Oncologist, the patient presented multiple large scalp metastases. The largest mass (10 cm) appeared at the right fronto-temporal region of the scalp: it was crusted and easily bleeding. Other slightly smaller lesions with the same characteristics were localized at the left pre-auricular region, at the left temporal region and at the nape. A thermoplastic mask for immobilization was made up, and a CT scan of the head and neck was performed for radiotherapy (RT) planning. The largest and bleeding right fronto-temporal metastasis was contoured as Gross Tumor Volume (GTV); the Planning Target Volume (PTV) was obtained through the 5 mm expansion of the GTV. No signs of infiltration of deep subcutaneous tissues or bone were detected at the planning CT. A total dose of 30 Gy in 10 fractions was prescribed. Six 6 MV non-coplanar photon beams tangent to the skin were used for irradiation (Fig. 4). Organ-at-risk (brain, lens, eye) constraints were respected.

During RT course, the treated mass stopped bleeding and showed a progressive shrinkage from 10 to 6–7 cm. The skin became increasingly crusted and rough. The patient did not showed acute treatment-related side effects at physical examination. Unfortunately, the other untreated masses continued to grow and some of them began to bleed.

After completion of RT, a second line chemotherapy with folinic acid, fluorouracil and irinotecan (FOLFIRI) was started but soon discontinued for thrombocytopenia.

The patient then was again directed to the radiation oncologist for a re-evaluation. Now the largest lesions, heavily bleeding, appeared at the right scapular region and at the back of the left thoracic wall. Both were approximately 10–12 cm diameter, heavily bleeding, partially necrotic and crusted. The quality of life of the patient was greatly compromised: she could not take lying down, so she couldn’t sleep at night and have a refreshing rest. The previously treated lesion of the scalp now was about 4 cm, dry, crusted and faintly adherent to the underlying skin.

A new planning CT was performed for RT. This time the patient was positioned prone. The two masses of the back were contoured separately as two GTVs, and two distinct PTVs were obtained adding a 5 mm margin. For irradiation, we used the same technique as the previous treatment (two and three tangent 6 MV photon beams). Given the multiplicity of the lesions, the clinical condition of the patient and the ongoing chemotherapy, we decided to administer a total dose of 20 Gy in 5 fractions to each target simultaneously.

The response to this course of RT was quick and surprising. Already after the first two fractions, both lesions stopped bleeding and appeared reduced in volume. At the end of RT course, they were all crusted and more movable. The necrotic appearance vanished.

After a week, the two masses appeared even smaller and drier and less vascularized due to the hemostatic effect of RT. No acute/subacute side effects were reported.

**Fig. 5:**
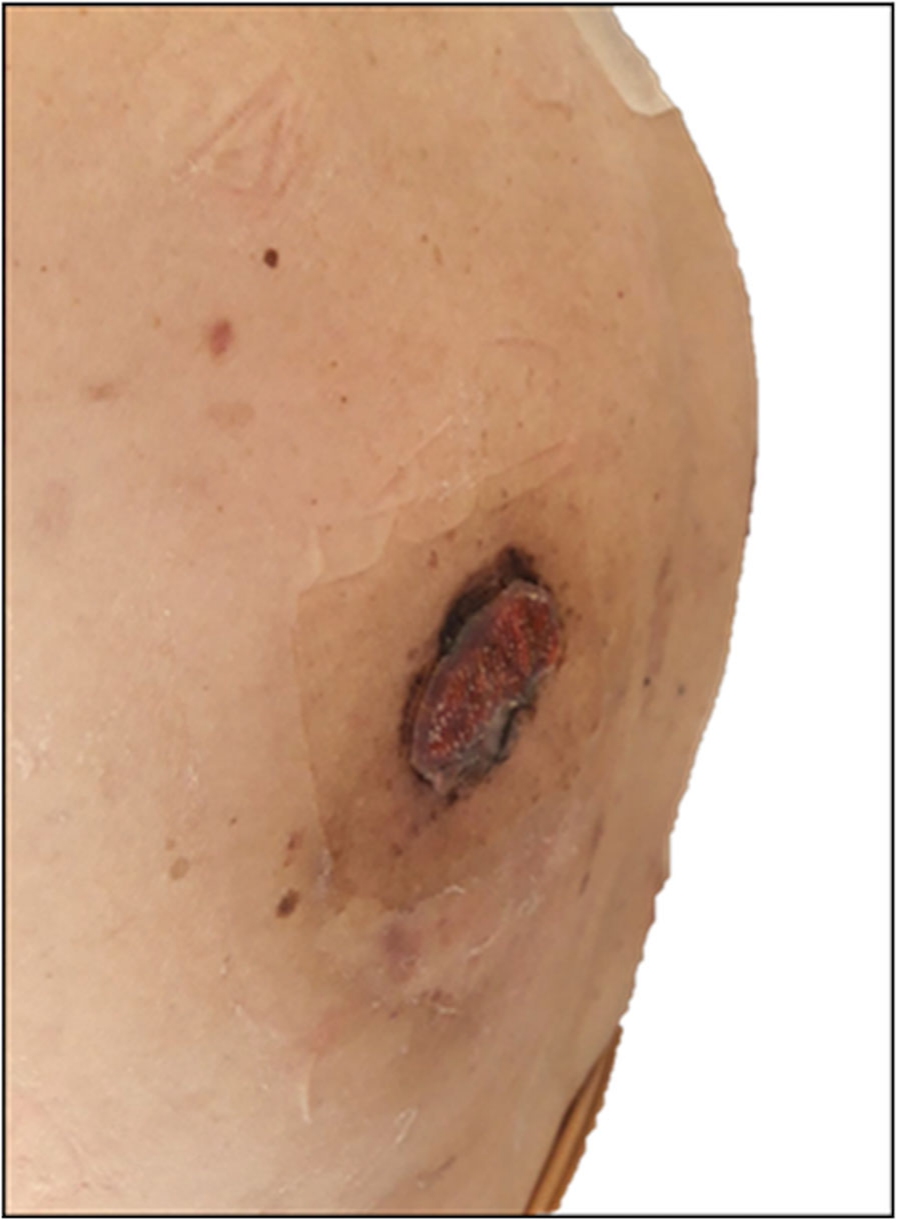
Clinical response of the irradiated skin lesion at the right scapular region

After 2 weeks, both lesions had detached from the skin, leaving two small flat crusty areas of a few centimeters (Fig. 5). The patient agreed that she could have started to relax and to rest better, obtaining a beneficial impact on her quality of life.

A few days later, the patient restarted chemotherapy with FOLFIRI, interrupted after two cycles because of hematological toxicity. During the chemotherapic treatment, the skin lesions showed an overall diffuse reduction of volume. After discontinuation of chemotherapy, a new progression of the skin metastases, particularly at the level of the left temple and nape, occurred.

The conditions of the lesions treated with RT, however, remained stationary. At the restaging CT the tumor visceral metastases showed a substantial stability. Unfortunately, the patient gradually developed a cachexia that led her to death about 2 months after completion of RT.

## Discussion and conclusions

Cases of skin (cutaneous/subcutaneous) metastases derived from neuroendocrine tumors of the lung [3–8], gastrointestinal [2, 9–11] and genitourinary tract [12–15] – as well as of other origins or with unknown primary tumor site [16–18] – have been exceptionally reported. In line with observations and revisions by Amorim et Al. [16], we remark that the detection of the primary tumor site in metastatic neuroendocrine tumors is a crucial question to improve the choice of therapeutic strategy. Anyhow, for patients with confirmed metastatic disease, systemic therapies and palliative cares of symptoms are the most common approaches [19]. Our overview of previous published cases with skin metastases from neuroendocrine tumors [3, 4, 6–8, 10–12, 14, 16–18] confirms this finding.

In published works, (sub) cutaneous metastases from neuroendocrine tumors are reported as single or multiple nodules, with typical location in the scalp/cephalic district and/or trunk, painless in the majority of cases [16]. Zuetenhorst et Al. [20] investigated the pathogenesis of pain which can be eventually associated to these skin metastases and reported that no differences in neuroinvasion, angioinvasion or mitosis between painful and non-painful metastases could be demonstrated. Authors also observed the poor pain-control using analgesics, irradiation or systemic treatments (e.g. interferon or chemotherapy), experiencing that local excision was the only successful treatment option [20].

The role of RT in the treatment of skin metastases is not well defined and few cases are reported in literature. Data regarding the optimal dose are lacking. Mak et al. [21] reported a significant response to RT of painful and bleeding cutaneous metastases from prostate cancer with a dose of 18 Gy in 3 fractions. A meta-analysis [22] reported response and recurrence rates for skin metastases after local palliative therapies (including radiation therapy, brachytherapy, surgery, electro-chemotherapy, topical, intralesional, or photodynamic therapy). Patients treated with RT (n.120), alone or in combination with other local therapies, showed a complete response rate of more than 60% of cases. However, only two prospective trials were included in the analysis and the primary tumor was breast cancer or melanoma in more than 90% of cases [22].

In our presented rare case of skin metastases from a small cell PNEC, we performed RT for growing and bleeding lesions using two typical palliative fractionation schedules (30 Gy/10 fractions and 20 Gy/5 fractions). Even if these treatments were performed with a purely palliative intent and dosage, we observed a good and unexpected local response of the treated lesions, which was also maintained with chemotherapy. Both the RT courses were well-tolerated and patient’s quality of life resulted improved.

A recent systematic review on the role of RT in the treatment of gastro-entero-pancreatic neuroendocrine tumors [19] pointed out the lack of high-quality evidences regarding the overall role of RT both in the treatment of primary tumor and metastatic site. Nevertheless, authors confirmed the relationship between RT and reasonably high rates of local control and symptoms palliation. An accurate selection of patients who are candidates for RT, as well as more consistent results from novel fractionation schedules adopted in different clinical scenarios [19] and further knowledge of the synergistic effect of RT and new systemic therapies [19], could support an effective application of RT and improve patients’ outcomes.

Prognosis of NETs with skin metastases remains poor. Our patient and the most reported cases [4–6, 8, 9, 11, 12, 15, 17, 18] died few months after diagnosis. According to our experience, personalized palliative approaches and a multidisciplinary patient’s evaluation (as recommended by international guidelines) lead to improve patient’s outcome and quality of life. Furthermore, our multidisciplinary approach reduced the overall time for the completion of diagnostic path and enabled a better management of iatrogenic toxicity.

In conclusions, the rarity of cases with skin metastases from PNETs requires efforts to improve the choice of the optimal palliation strategy. In selected patients with bleeding and/or growing lesions, RT could be proposed to reduce local symptoms. Because the most common palliative fractionation schedules are also usually well-tolerated, RT could successfully improve patients’ quality of life. Nevertheless, an integrated multidisciplinary team evaluation aimed at personalized approaches is necessary in the therapeutic management of similar cases with a poor prognosis.

## Data Availability

Data sharing is not applicable to this article as no datasets were generated or analyzed.

## Abbreviations

PNEC: Pancreatic neuroendocrine carcinoma
(P)NET(s): (Pancreatic) neuroendocrine tumor(s)
CT: Computer Tomography
NSE: Neuron Specific Enolase
RT: Radiotherapy
GTV: Gross Tumor Volume
PTV: Planning Target Volume
FOLFIRI: Folinic acid, Fluorouracil and Irinotecan

## References

[1] Rindi G, Klimstra DS, Abedi-Ardekani B (2018). A common classification framework for neuroendocrine neoplasms: an International Agency for Research on Cancer (IARC) and World Health Organization (WHO) expert consensus proposal. Mod Pathol.

[2] Laschinger ME, Naga L, Gaspari AA (2018). Cutaneous metastases of pancreatic neuroendocrine carcinoma. G Ital Dermatol Venereol.

[3] Linhas R, Tente D, China N, Conde S, Barroso A (2018). Subcutaneous metastasis of a pulmonary carcinoid tumor: a case report. Medicine (Baltimore).

[4] Falto-Aizpurua L, Seyfer S, Krishnan B, Orengo I (2017). Cutaneous metastasis of a pulmonary carcinoid tumor. Cutis.

[5] Mestre T, Rodrigues AM, Cardoso J (2015). Pulmonary large-cell neuroendocrine carcinoma presenting as multiple cutaneous metastases. J Bras Pneumol.

[6] Pajaziti L, Hapçiu SR, Dobruna S, Hoxha N, Kurshumliu F, Pajaziti A (2015). Skin metastases from lung cancer: a case report. BMC Res Notes.

[7] Ishida M, Iwai M, Kagotani A, Iwamoto N, Okabe H (2014). Cutaneous metastasis from pulmonary large cell neuroendocrine carcinoma in the scalp. Int J Clin Exp Pathol.

[8] Brinkman D, Roche L, Ullah K, O'Connor TM (2013). Multiple cutaneous nodules as the presenting sign of small cell lung cancer. BMJ Case Rep.

[9] Białecki M, Białecka A, Męcińska-Jundziłł K, Adamska U, Kasperska A, Czajkowski R (2018). Imaging in a rare case of neuroendocrine tumour with skin metastases. Pol J Radiol.

[10] Shin WY, Lee KY, Ahn SI, Park SY, Park KM (2015). Cutaneous metastasis as an initial presentation of a non-functioning pancreatic neuroendocrine tumor. World J Gastroenterol.

[11] Wang SM, Ye M, Ni SM (2014). Multiple scalp metastases from colonic neuroendocrine carcinoma: case report and literature review. BMC Cancer.

[12] Cokmert S, Demir L, Doganay L, Demir N, Kocacelebi K, Unek IT, Gezer E, Kilic K, Alakavuklar M (2014). Large cell neuroendocrine carcinoma of the ovary and its skin metastases: a case report and review of the literature. West Indian Med J.

[13] Devnani B, Kumar R, Pathy S, Phulware RH, Mathur S, Kumar L. Cutaneous metastases from neuroendocrine carcinoma of the cervix-An unusual metastatic lesion from an uncommon malignancy. Curr Probl Cancer. 2018. 10.1016/j.currproblcancer.2018.04.004. 10.1016/j.currproblcancer.2018.04.004 29937242

[14] Lee WJ, Kim CH, Chang SE, Lee MW, Choi JH, Moon KC, Koh JK (2009). Cutaneous metastasis from large-cell neuroendocrine carcinoma of the urinary bladder expressing CK20 and TTF-1. Am J Dermatopathol.

[15] Kaplan M, Atakan IH, Bilgi S, Inci O (2007). Case report: subcutaneous metastasis from small cell carcinoma of the prostate. Int Urol Nephrol.

[16] Amorim GM, Quintella D, Cuzzi T, Rodrigues R, Ramos-e-Silva M (2015). Cutaneous metastasis of neuroendocrine carcinoma with unknown primary site: case report and review of the literature. Case Rep Dermatol.

[17] Shin MK, Choi CM, Oh YJ, Kim NI (2011). CK20 positive large-cell neuroendocrine carcinoma presenting with skin metastases. Ann Dermatol.

[18] Garcia A, Mays S, Silapunt S. Metastatic neuroendocrine carcinoma in the skin. Dermatol Online J. 2017;23(1):8. 28329472

[19] Chan DL, Thompson R, Lam M (2018). External beam radiotherapy in the treatment of gastroenteropancreatic neuroendocrine tumours: a systematic review. Clin Oncol.

[20] Zuetenhorst JM, van Velthuysen ML, Rutgers EJ, Boot H, Taal BC (2002). Pathogenesis and treatment of pain caused by skin metastases in neuroendocrine tumours. Neth J Med.

[21] Mak G, Chin M, Nahar N, De Souza P (2014). Cutaneous metastasis of prostate carcinoma treated with radiotherapy: a case presentation. BMC Res Notes.

[22] Pratt DE, Gordon Spratt EA, Wu S, DeRosa A, Lee NY, Lacouture ME, Barker CA (2014). Efficacy of skin-directed therapy for cutaneous metastases from advanced cancer: a meta-analysis. J Clin Oncol.

